# Trends in Pediatric In-Training Exam Scores and Association With Clinical Encounters Across the COVID-19 Pandemic

**DOI:** 10.7759/cureus.96299

**Published:** 2025-11-07

**Authors:** Priyanka Rao, Katherine Jordan, Heather Burrows, Lauren Helms, Rebecca Perin, Michael Andrew Crawford, Daniel Kang, Andrew Morgan, Margaret Kihlstrom, Eric Zwemer

**Affiliations:** 1 Pediatrics and Child Health, University of North Carolina at Chapel Hill, Chapel Hill, USA; 2 Pediatrics, University of North Carolina at Chapel Hill, Chapel Hill, USA; 3 Pediatrics, University of Michigan, Ann Arbor, USA; 4 Pediatrics, University of Arkansas for Medical Sciences, Little Rock, USA; 5 Pediatrics, University of California Irvine School of Medicine, Irvine, USA; 6 Internal Medicine, University of North Carolina at Chapel Hill School of Medicine, Chapel Hill, USA; 7 Pediatric Critical Care Medicine, University of North Carolina at Chapel Hill, Chapel Hill, USA

**Keywords:** clinical exposure, covid-19, education, in training exam, pediatric residency

## Abstract

Pediatric In-Training Exam (ITE) scores dropped during the pandemic and prior work showed this drop was unrelated to clinical volume. It remained unclear how ITE scores would change post pandemic peak. We compared ITE scores with data on patient notes for five classes of pediatric residents matriculating from 2018 through 2022 at four residency programs which included a total of 391 residents. Post-graduate year (PGY) 2 ITE was 11.6 points lower for residents starting in 2020 compared to other classes (95% CI 8.3-14.8 points). While number of inpatient admission notes (p = 0.012) and progress notes (p = 0.0044) were associated with statistically significant gains in PGY2 ITE scores, clinical note volume only accounted for a small amount of the cohort effect (4.8%). There are some types of clinical encounters that are statistically associated with ITE score gains and while there was partial recovery of scores compared to pre-pandemic cohorts, total PGY3 scores remain lower than the 2018 class.

## Introduction

The pediatric In-Training Examination (ITE) is a standardized examination taken in July of each year of residency, and programs use the ITE to assess resident knowledge, monitor progress in knowledge acquisition, and predict future success at passing the initial certifying examination [1]. A prior study examined ITE scores and clinical volume, finding that both ITE scores and clinical encounters decreased during the COVID-19 pandemic for residents matriculating between 2018-2020, representing classes before and at the beginning of the pandemic. However there was no association between total clinical encounters and the change in ITE score from post-graduate year (PGY) 1 and PGY2 years [2]. The learning environment changed dramatically during the pandemic: clinical exposure declined, didactic learning switched to virtual formats, and measures of psychological distress and burnout increased [3-6]. As the learning environment evolved post-pandemic, we sought to expand upon the prior study by examining the trajectory of resident ITE scores and clinical encounter numbers for classes matriculating between 2018-2022, covering periods before, during, and after the pandemic’s peak. With its global disruption, and subsequent recovery, the COVID-19 pandemic offers us a chance to study on a broad level the ability of residency training programs to quickly adjust and adapt to changes and challenges in the learning environment. We hope that findings from our study might provide insight for program directors as they adapt to changes all programs periodically experience on a more local level.

## Materials and methods

**Figure 1:**
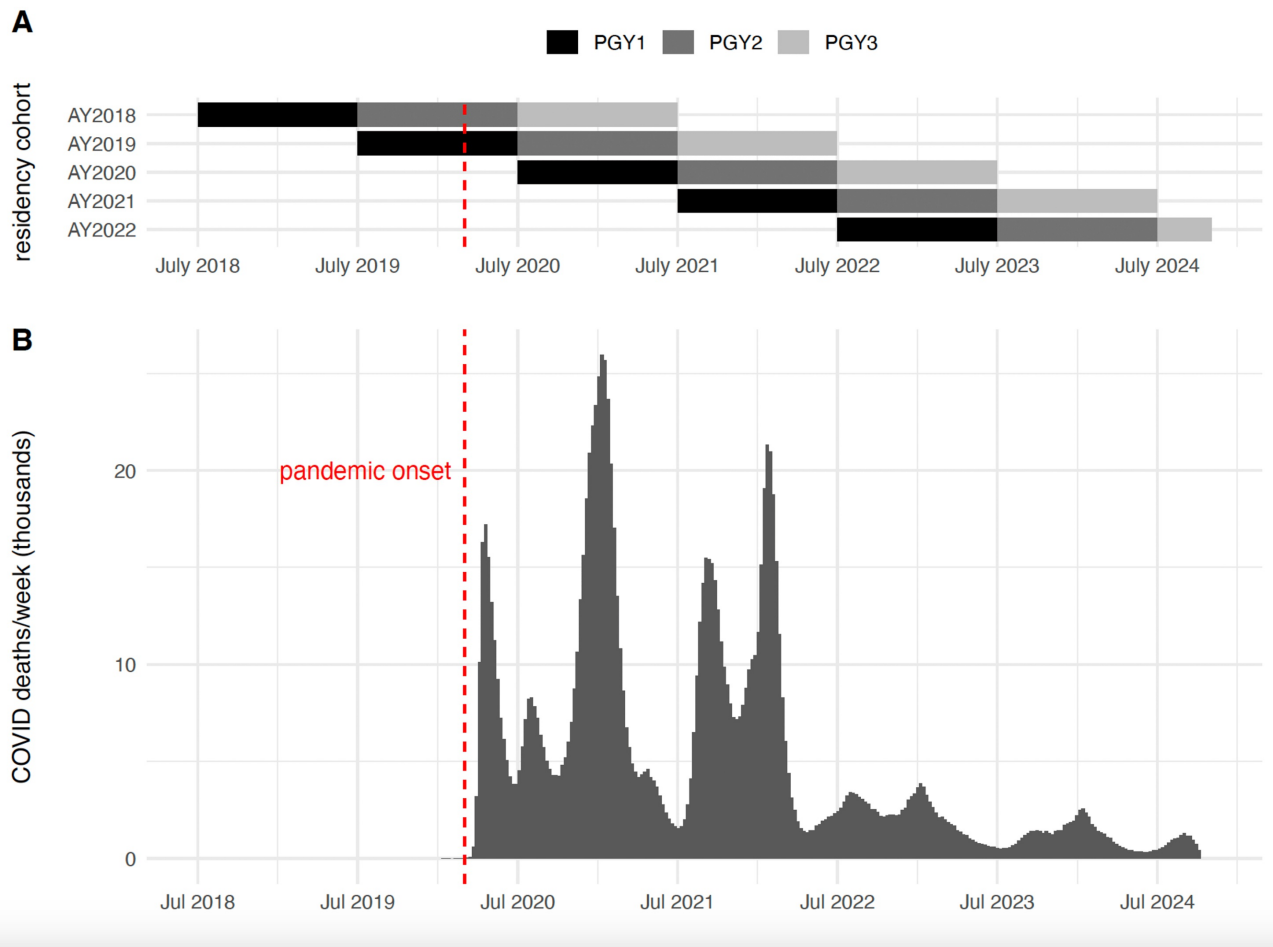
Temporal relationships between residency cohorts in this manuscript (panel A) and COVID-19 pandemic intensity, summarized by total weekly COVID-19 deaths in the US (panel B). Note that the plot is truncated at October 2024. Figure created by authors using CDC COVID Data Tracker data (https://covid.cdc.gov/covid-data-tracker/) PGY: post-graduate year, AY: academic year

This was a multi-site retrospective cohort study. Subjects (n = 391) were residents matriculating between 2018 and 2022 at four pediatric residency programs: the University of North Carolina (UNC), the University of Michigan, the University of Arkansas (UAMS), and the University of California at Irvine/Children’s Health of Orange County (UCI/CHOC) (Figure 1).

PGY1, PGY2, and PGY3 ITE scores, United States Medical Licensing Examination (USMLE) Step 2 CK scores, and, if applicable, Comprehensive Osteopathic Medical Licensing Examination (COMLEX) scores, were provided by residency leadership at each program. (The 2022 cohort had not taken their PGY3 ITE score at the time of this study.) COMLEX scores were used for only a small minority of subjects (14/391, 3.5%) and were converted to Step 2 CK via percentile matching using a published formula [7]. 

The number of clinical notes written per resident per year was used as a proxy for the number of clinical encounters in which they participated. We tabulated outpatient notes, inpatient progress notes, inpatient history and physical notes, and emergency room notes using the EPIC^TM^ (Epic Systems, Verona, WI, USA) electronic medical record (EMR) report function, if applicable. This captures only the notes completed at the primary (academic) clinical site for each residency program. As such, notes completed during approximately 25% of clinical time at a community site in one residency, and 8% in another residency, could not be captured. To account for these differences, note counts were standardized to zero mean and unit variance within programs. Each unit change in the standardized score thus represents one standard deviation change in number of notes written. 

**Figure 2:**
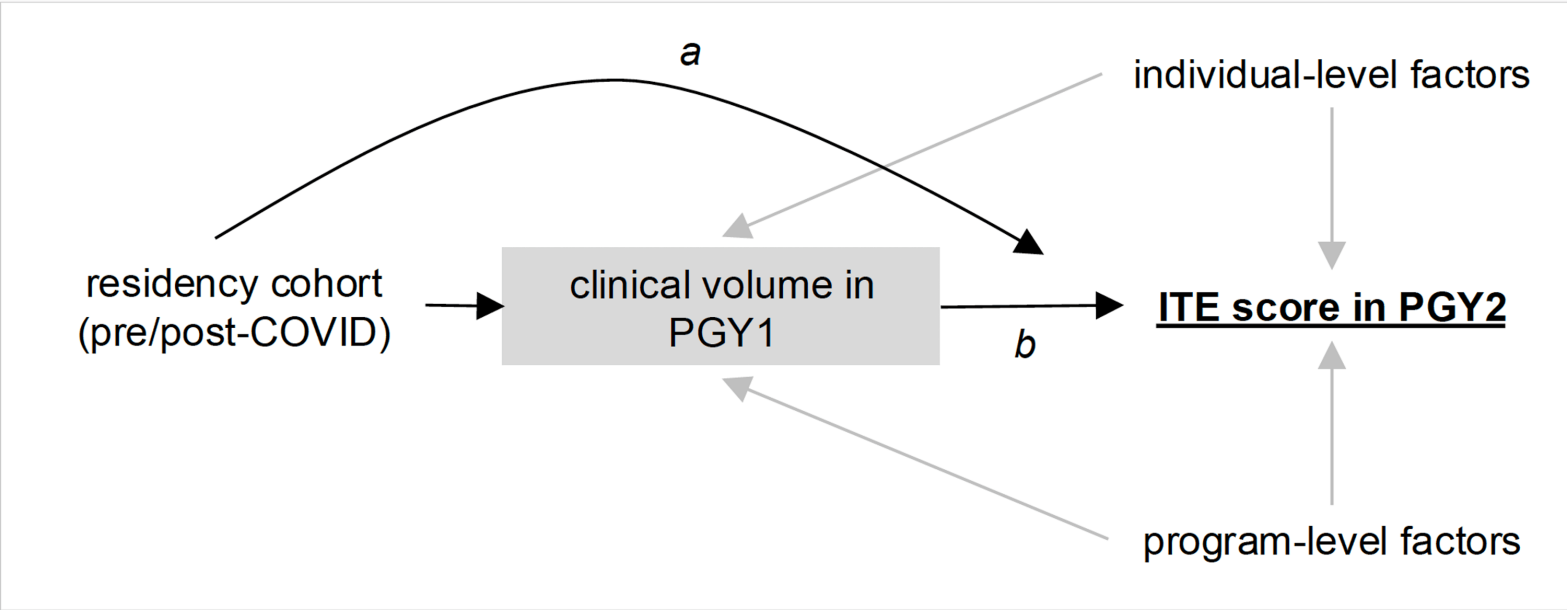
Relationship between covariates, outcome (underlined text) and exposure of interest (grey box) depicted as a directed acyclyic graph (DAG). Grey arrows indicate measured confounders. Black arrows show causal effects of interest. (a) Direct effect of pandemic cohort status on ITE scores. Assumed to encompass any and all unmeasured factors besides the exposure of interest, clinical volume (grey box). (b) The causal effect of interest. PGY: post-graduate year, ITE: In-Training Exam

Associations between ITE scores and covariates of interest were analyzed with linear models in R v4.1.1 (R Foundation for Statistical Computing, Vienna, Austria). Residency program and matriculation year (“cohort”) were treated as fixed effects. Marginal means and contrasts were estimated using functions from the emmeans package v1.7.2. To formally test the hypothesis that decline in PGY2 ITE scores across the pandemic was due to decrease in clinical volume, we performed a mediation analysis [8]. The directed acyclic graph (DAG) (Figure 2) describes assumed causal relationships between the covariates in this study and the outcome (PGY2 ITE score). The “direct effect” of pandemic cohort status on ITE scores is (a) and is assumed to encompass any and all unmeasured factors besides the exposure of interest, clinical volume (grey box). The causal effect of interest is (b). Estimates of causal effects were obtained using the mediation package v4.5.0. This study was reviewed by each program’s Institutional Review Board and deemed exempt or approved for study (UNC: IRB 22-0362, University of Michigan: IRB HUM00216927, UAMS/ACH: IRB 274331, UCI/CHOC: IRB 220334). 

## Results

Our study included 391 total residents across the four pediatric residency programs with class cohorts ranging from 13 to 26 (Table 1).

**Table 1:** Number of residents per year and program Program A, B, C, D: Each letter represents one of the individual residency programs included in our study. Programs to remain anonymous to protect individual resident anonymity. AY: academic year

	AY 2018	AY 2019	AY 2020	AY2021	AY2022	Sum
Program A	21	21	21	22	21	106
Program B	16	13	16	21	16	82
Program C	14	16	17	17	17	81
Program D	22	26	25	24	24	122
Sum	73	76	79	84	79	391

**Figure 3:**
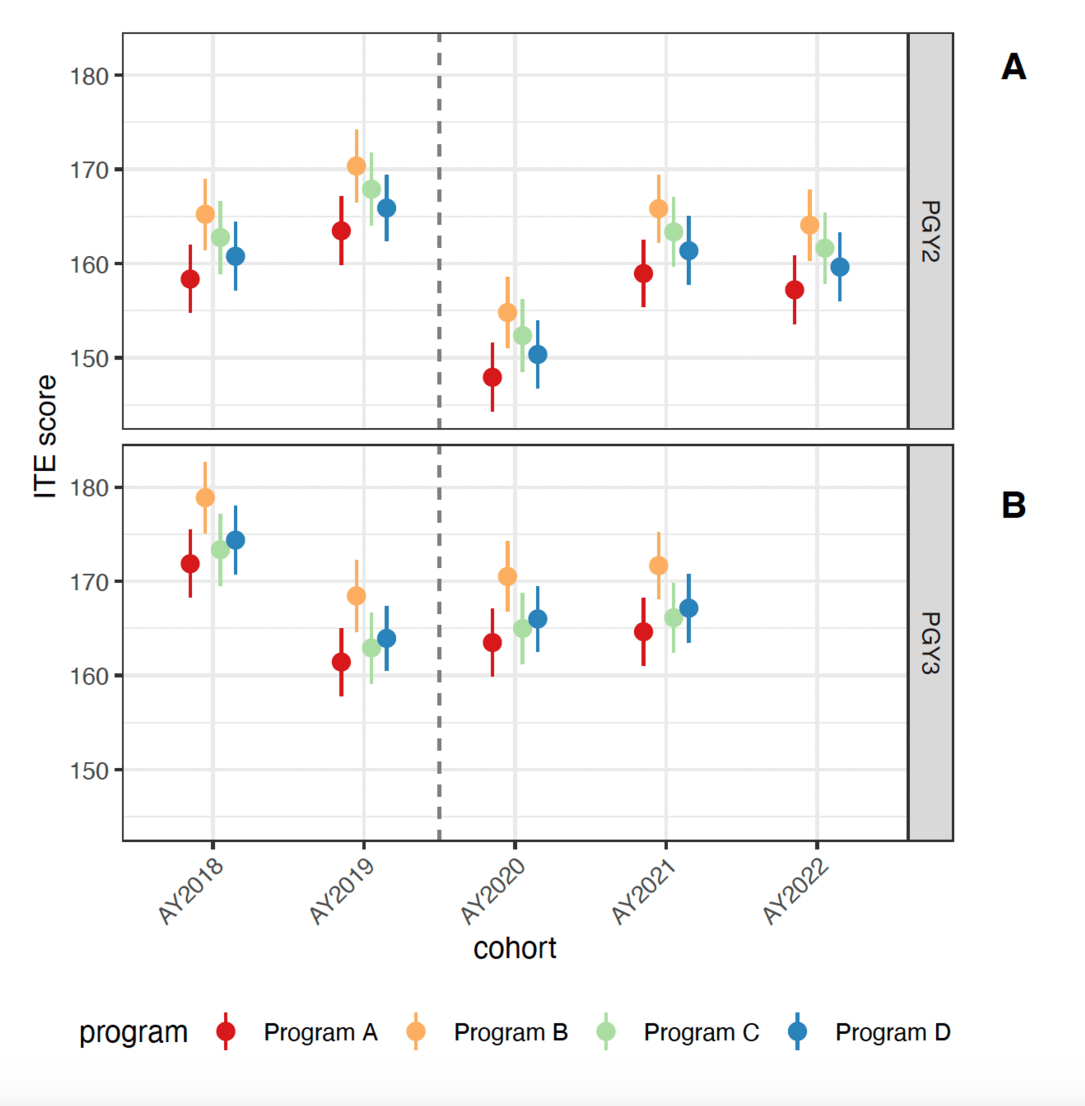
Estimated mean ITE scores by residency program and cohort, for PGY2 (A) and PGY3 (B). Error bars represent 95% confidence intervals for group means. Note that PGY3 scores are not yet available for the 2022 cohort. Program A, B, C, D: Each letter represents one of the individual residency programs included in our study. Programs to remain anonymous to protect individual resident anonymity. PGY: post-graduate year, AY: academic year, ITE: In-Training Exam

Only 1.3% of ITE scores were missing. After controlling for ITE score in PGY1, ITE scores in PGY2 were, on average, 11.6 points (95% CI 8.3 - 14.8 points) lower for individuals who began residency in 2020 compared to those who began residency in the two years before or after (Figure 3).

For the PGY3 ITE, scores appear to have dropped starting with the 2019 cohort, with the 2018 cohort averaging 8.7 points higher than all other cohorts (95% CI 5.5 - 11.8 points). The mean difference between the PGY3 ITE score for the 2020 cohort and the 2019 and 2021 cohorts, however, was not significant (0.5 points, 95% CI -2.8 - 3.7 points; Figure 3). These patterns were qualitatively similar across the four residency programs in our study (Figure 3). Using change in ITE score compared to baseline in PGY1, rather than absolute scores, yielded similar results. 

**Figure 4:**
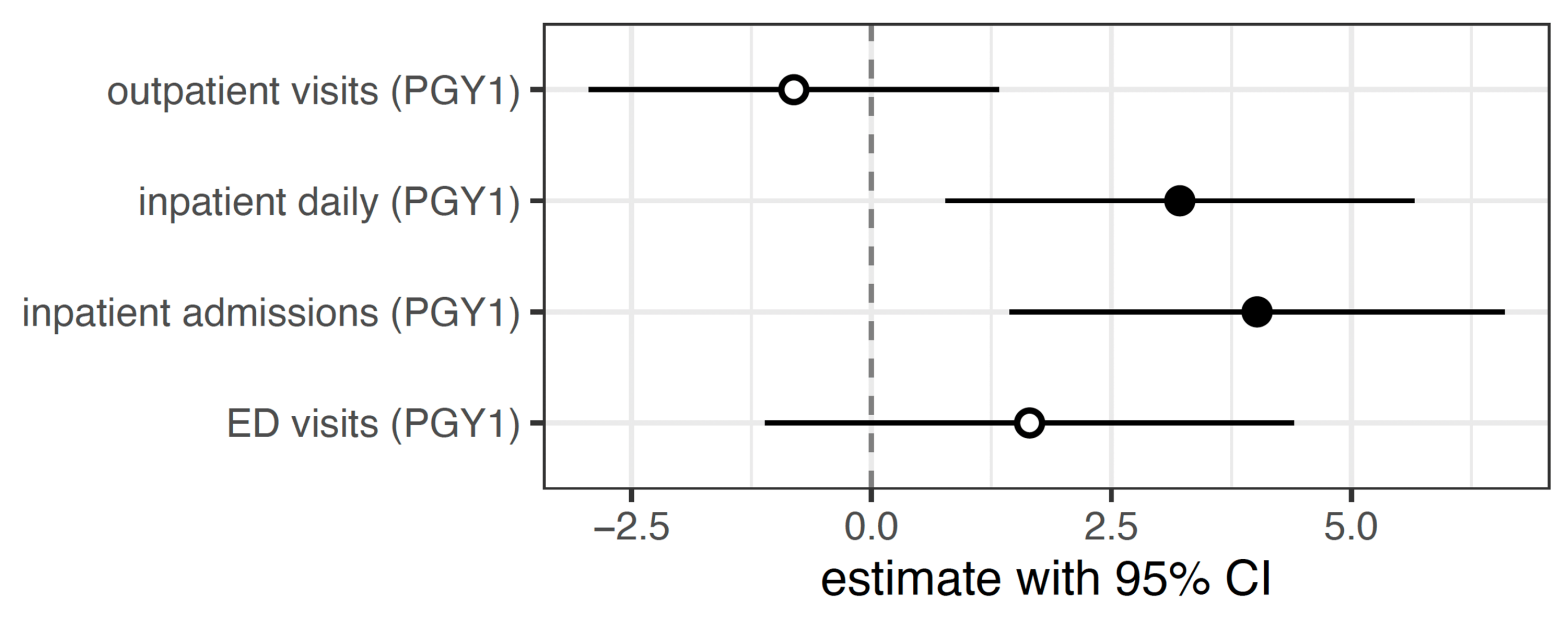
Effect of clinical volume on ITE score in PGY2. Units are ITE exam points per 1 standard deviation change in number of patient encounter of each type. PGY: post-graduate year, ITE: In-Training Exam

When evaluating the volume of clinical exposure and change in ITE score from PGY1 to PGY2, the number of inpatient admission notes (p = 0.012) and inpatient progress notes (p = 0.0044) were associated with statistically significant gains in ITE score from PGY1 to PGY2 (Figure 4).

This was not true for outpatient (p=0.89) or ED notes (p=0.29). However, mediation analysis suggests that little of the cohort effect is explained by note volume: the average mediated casual effect of inpatient admission notes is just 0.8 ITE score points (95% CI 0.01 - 1.6 points) per 1 standard deviation change in number of notes written, only 4.8% of the total cohort effect [8]. Overall, ITE performance showed partial recovery post-pandemic, but scores have not returned to pre-2019 levels.

## Discussion

We examined the trajectory of ITE scores for residents whose training overlapped the COVID-19 pandemic [2]. We found that PGY2 scores dropped significantly compared to peer residents but partial recovery was seen by PGY3. Taken together, these results suggest that the effect of the pandemic on knowledge acquisition early in residency has been attenuated, but some disparities remain. 

Adequate volume and variety of clinical exposure are vital for resident education. There were notable changes in both work hours and patient encounters in multiple specialties during the pandemic. A study examining work hours for over 1100 residents from multiple residency programs at three Mayo Clinic-affiliated campuses across the country found significantly decreased work hours in 19 out of 43 programs at the height of the pandemic (April 2020) with an associated increase in hours worked from home [9]. In their study, pediatrics residencies did not have a statistically significant change in work hours. In a large urban pediatric residency program, however, pediatric interns had a significant decrease in patient encounters on the hospital medicine and intensive care step-down unit teams, along with a decrease in number of shifts worked [10]. Another survey of pediatric residency program directors reported significant decreases in inpatient encounters and increases in telemedicine encounters, alongside increased absences due to the pandemic [11]. This echoes another study finding that the number of clinical encounters changed during the pandemic, although the magnitude of the change and distribution of impact across inpatient versus outpatient contexts varied between programs [2]. While we found that inpatient daily progress notes and admission notes statistically correlated with a gain in ITE scores, clinical encounter numbers explain only a small portion of the cohort differences. 

Our analysis showed that inpatient, but not outpatient or emergency, clinical work was associated with a small improvement in ITE scores. We postulate that there are qualitative differences between the settings in the type of learning that is experienced by residents. When examining this finding through Kolb’s experiential learning theory, the inpatient setting may be better positioned for experiential learning. Kolb’s theory postulates that in order to learn from experiences, we need to have a concrete experience, reflect on it, conceptualize what we could do differently, and then actively experiment with that knowledge [12]. In many training programs, the inpatient setting best supports this experience, where residents are able to see patients daily as their diagnosis and response (or not) to treatment unfolds. In many outpatient settings, residents are not scheduled in a way to follow up the same patients to observe their response to treatment [13]. Similarly, in the emergency room, the fast pace and shift nature of the work mean that residents may not see the conclusion or follow-up for patients. However, it is also possible that we will see shifts in the types of encounters most important for knowledge tested by the Pediatric ITE and Board Certification Exams in future studies, as the content specifications changed in 2024 to include a heavier emphasis on preventative well-child care and mental health care. 

Many factors other than patient encounters and work hours likely affected the learning environment during the pandemic - changes in didactic educational sessions with many being virtual, changes in the psychosocial components of the in-person learning environment, and increased mental health stressors [14]. Pediatric residency program directors nationally reported that the pandemic had negative effects on pediatric resident trainees, with concern that trainees were not being adequately prepared for roles as senior residents [15]. In a national survey at the onset of the pandemic, pediatric residents reported that many aspects of their training were negatively affected by the COVID-19 pandemic, including change in type and amount of clinical work, personal wellness, burden of caring for dependents with disruption in school and childcare, and negative impact on educational activities such as balance of education and service, conferences and scholarly work [16]. It is likely that these global changes to the learning environment played a larger role in changes in preparedness reported by program directors and the decrease in ITE scores noted in our study. 

Our study has several limitations. The literature reports many factors may have contributed to changes in resident learning during the pandemic, including changes in the format and attendance of didactic sessions [17-19]. We did not have retrospective access to such conference data for the programs in this study. Additionally, we were not able to study the complex social and environmental aspects that pediatric residents reported contributed to their learning environment during the pandemic [16]. The small number of programs in our study is another limitation. While geographically diverse, this sample does not encompass the breadth of training environments nationwide, and in particular, includes only academic and not community-based programs. Finally, while it is known that pediatric initial certifying exam pass rates declined across the years of the pandemic, we chose not to examine the relationship between ITE scores and certifying exam performance [20]. The small sample size of our study would limit the power of such an analysis and could risk compromising trainee confidentiality. 

## Conclusions

In conclusion, this follow-up study demonstrated that while the 2020 cohort performed worse on PGY2 ITE compared to the cohorts before and after, they had equivalent performance on PGY3 ITE compared to post-pandemic peers. While there was variability in clinical encounter exposures that correlated with ITE score, this does not explain variation between cohorts. We believe the cause of the drop in ITE scores in 2020 was likely multifactorial and are encouraged that ITE scores have since recovered in subsequent cohorts. Future studies should examine how these cohorts performed on their initial certifying examinations. Residency programs should consider ways to support residents and their learning in cohorts who are affected by other types of life-altering events, with encouragement from this study that trainee education demonstrated resilience following the peak of the COVID-19 pandemic. Programs should prioritize strategies that preserve experiential learning and trainee wellness during disruptions in the traditional academic environment.
